# Detection of respiratory syncytial virus and rhinovirus in healthy infants

**DOI:** 10.1186/s13104-015-1695-6

**Published:** 2015-11-25

**Authors:** Kohei Hasegawa, Rachel W. Linnemann, Vasanthi Avadhanula, Jonathan M. Mansbach, Pedro A. Piedra, James E. Gern, Carlos A. Camargo

**Affiliations:** Department of Emergency Medicine, Massachusetts General Hospital, Harvard Medical School, 125 Nashua Street, Suite 920, Boston, MA 02114 USA; Division of Pediatric Pulmonology, Department of Pediatrics, Massachusetts General Hospital, Harvard Medical School, Boston, MA USA; Departments of Molecular Virology and Microbiology, Baylor College of Medicine, Houston, TX USA; Departments of Molecular Virology and Microbiology and Pediatrics, Baylor College of Medicine, Houston, TX USA; Department of Medicine, Boston Children’s Hospital, Harvard Medical School, Boston, MA USA; Departments of Pediatrics and Medicine, University of Wisconsin School of Medicine and Public Health, Madison, WI USA

**Keywords:** Respiratory syncytial virus, Rhinovirus, Healthy infants, Asymptomatic, Prevalence

## Abstract

**Background:**

Despite the research importance of rhinovirus detection in asymptomatic healthy infants, the literature remains sparse.

**Objective:**

To investigate the prevalence of respiratory syncytial virus (RSV) and rhinovirus (and its species).

**Methods:**

We conducted a cross-sectional study of 110 healthy, non-hospitalized infants without acute illness at an academic medical center from November 2013 through May 2014. We tested nasal swab specimens by using polymerase chain reaction and genetic sequencing.

**Results:**

Overall, the median age was 3.8 months (IQR 2.0–5.1 months), 56 % were male, and 90 % were born >37 weeks. RSV was detected in nasal swabs from infants (1.8 %). By contrast, rhinovirus was detected in nasal swabs from 16 infants (14.5 %). Molecular typing assay revealed rhinovirus species: six rhinovirus-A (5.5 %), one rhinovirus-B (0.9 %), eight rhinovirus-C (7.3 %), and one untypeable (0.9 %).

**Conclusions:**

In this cross-sectional study of healthy, community-based infants, RSV was rare (<2 %) in nasal swabs, while rhinovirus was detected in 14.5 % with a predominance of rhinovirus-A and -C. These finding are important for understanding the clinical significance of rhinovirus detection among infants hospitalized for bronchiolitis.

## Background

Respiratory syncytial virus (RSV) and rhinovirus are the most frequently detected viral pathogens in infants with bronchiolitis, a leading cause of emergency department visit [1, 2] and hospitalization for United States infants [3]. Although it has long been the conventional wisdom that the infectious etiology of bronchiolitis does not affect outcomes, a growing number of studies have linked viral pathogens of bronchiolitis (e.g., rhinovirus) to long-term respiratory outcomes [4]. For example, studies reported that RSV bronchiolitis in infancy, especially when severe (e.g., requiring hospitalization) contribute to subsequent development of childhood asthma, while more recent research has focused on the potential role of rhinovirus bronchiolitis, particularly rhinovirus species C, in early life in asthma pathogenesis [4].

A causal role of RSV and rhinovirus in lower respiratory infection is supported by isolation of viruses from the respiratory tract of children. However, advances in viral detection techniques (e.g., polymerase chain reaction [PCR] testing) have enabled increased identification of RSV and rhinovirus, not only in infants with acute respiratory infection (ARI) but also in asymptomatic individuals [5, 6]. A systematic review reported, in the respiratory samples of asymptomatic individuals (all ages), a mean viral prevalence of 2.6 % for RSV and 15.1 % for rhinovirus [5]. This finding questions the clinical significance of rhinovirus detection in young children with bronchiolitis. However, only a few studies, with small sample sizes, have specifically examined community-based infants (aged < 1 year) without comorbidities and without a high risk for atopy [7, 8]. Additionally, the prevalence of different rhinovirus species in this population remains largely unclear. To address these knowledge gaps, we investigated the prevalence of RSV and rhinovirus (and its species) by using PCR and molecular typing assay on nasal swab specimens from healthy, non-hospitalized infants without acute illness.

## Methods

### Study design and participants

We conducted a cross-sectional study of healthy infants enrolled from a primary care group practice at Massachusetts General Hospital (Boston, Massachusetts) from November 2013 through May 2014. Inclusion criteria were infants aged < 1 year and gestational age > 34 weeks. In the 3 days before a scheduled clinic visit, families were screened via phone to determine interest and eligibility. Exclusion criteria included comorbidities (heart–lung disease, immunodeficiency, immunosuppression, or chronic gastrointestinal disorder); previous lower respiratory infection that resulted in an urgent clinic visit, emergency department visit, or hospitalization; any diarrheal illness lasting >24 h in the past week; any treatment with antibiotics in the past week; and current fever, ARI, or gastrointestinal illness. We defined current ARI and gastrointestinal illness based on the literature [9]. ARI was defined as two of the following symptoms for 1 day, or one of the following symptoms for two consecutive days: runny nose, stuffy or blocked nose, cough, fever, sore throat, or sneezing. Gastrointestinal illness was defined as: two or more looser than normal stools or any vomiting within 24 h. The institutional review board of Massachusetts General Hospital approved the study. Informed consent was obtained from the parents or guardians.

### Data and sample collection

Clinical data were collected from parents via structured interview and from medical record review. Data included infants’ demographic characteristics as well as medical, environmental, and family history. Nasal swabs were collected from the anterior nares using a standardized protocol. Both nares were swabbed with a single nylon, pediatric FLOQSwab (Copan, Brescia, Italy). The swab was then added to 2 mL of viral transport medium and frozen at −80 °C. Samples were shipped on dry ice to the laboratory at Baylor College of Medicine, where they were stored at −80 °C. Samples were extracted and cDNA was generated using random hexamers for RSV and gene specific primers for rhinovirus. Singleplex and duplex real time PCR were used to detect rhinovirus and RSV respectively. Details of rhinovirus primers and probes have been described elsewhere [10], and sequences of RSV primers and probes are available upon request. Next, rhinovirus-positive specimens were sent to University of Wisconsin. Rhinovirus species were identified by using molecular typing assay that targets a variable fragment in 5′ untranslated region of the viral genome flanked by highly conserved motifs [11].

### Statistical analyses

All analyses were performed using Stata SE 13.1 software (Stata Corp, College Station, TX). Patient demographic and clinical characteristics were compared by rhinovirus status, using Mann–Whitney, Chi Square, or Fisher exact tests, as appropriate.

## Results

Table 1 summarizes demographic and clinical characteristics of the 110 enrolled infants. Overall, the median age was 3.8 months (IQR 2.0–5.1 months), 56 % were male, 51 % were non-Hispanic white, and 90 % were born >37 weeks.

**Table 1:** Demographic and clinical characteristics of 110 healthy infants

Characteristics	All infants (n = 110)	Rhinovirus positive (n = 16)	Rhinovirus negative (n = 94)	P value
*Demographics characteristics*				
Age in months, median (IQR)	3.8 (2.0–5.1)	4.3 (3.6–5.8)	2.6 (1.9–4.5)	0.03
Sex				0.60
Male	62 (56)	10 (63)	52 (55)	
Female	48 (44)	6 (38)	42 (45)	
Race/ethnicity				0.80
Non-hispanic white	56 (51)	10 (63)	46 (49)	
Non-hispanic black	11 (10)	1 (6)	10 (11)	
Hispanic	20 (18)	3 (19)	17 (18)	
Other	23 (21)	2 (13)	21 (22)	
Insurance				0.30
Private	89 (81)	15 (94)	74 (79)	
Public	21 (19)	1 (6)	20 (21)	
*Home environment characteristics*				
Sibling in home	47 (43)	7 (44)	40 (43)	0.93
Day care attendance	16 (15)	7 (44)	9 (10)	0.002
Exposure to passive smoke in home	4 (4)	2 (13)	2 (2)	0.04
*Clinical characteristics*				
Gestational age				0.30
≥40 weeks	46 (42)	4 (25)	42 (45)	
38–39 weeks	53 (48)	10 (63)	43 (46)	
34–37 weeks	11 (10)	2 (13)	9 (10)	
Delivery mode				0.92
Vaginal delivery	70 (64)	10 (63)	60 (64)	
Cesarean section	40 (36)	6 (38)	34 (36)	
Primarily breast milk feeding from ages 0 to 3 months	84 (76)	12 (75)	72 (77)	0.99
Eczema	17 (15)	4 (25)	13 (14)	0.27

Data were expressed as numbers (percentages) unless otherwise indicated

RSV was detected in nasal swabs from only two infants (1.8 %). By contrast, rhinovirus was detected in nasal swabs from 16 infants (14.5 %) with a median cycle threshold value of 29 (IQR 26–34). Molecular typing assay revealed rhinovirus species: six rhinovirus-A (5.5 %), one rhinovirus-B (0.9 %), eight rhinovirus-C (7.3 %), and one untypeable (0.9 %). Infants with detected rhinovirus were older than those without (4.3 vs. 2.6 months; P = 0.03), and were more likely to attend daycare (43.8 vs. 9.6 %; P = 0.002) and be exposed to passive smoke in home (12.5 vs. 2.1 %; P = 0.04). There were no other significant differences in demographic or clinical characteristics between the two groups (Table 1).

In a sensitivity analysis, we limited our analysis to infants who were completely symptom-free at the time of the screening phone call and enrollment. In this subset of 96 infants, RSV was detected in one infant (1.0 %) and rhinovirus in 11 (11.5 %). Molecular typing assay revealed six rhinovirus-A (6.3 %), no rhinovirus-B, four rhinovirus-C (4.2 %), and one untypeable (1.1 %).

## Discussion

In this study of 110 healthy, community-based infants, RSV was rarely detected (1.8 %). By contrast, rhinovirus was detected in the nasal swab specimens from 14.5 % of healthy infants, with predominance of rhinovirus-A and -C. When limiting our analysis to those who were completely asymptomatic at screening and enrollment, the rhinovirus prevalence decreased to 11.5 %.

Despite the research importance of rhinovirus (overall and species) detection in asymptomatic “healthy” infants, the literature remains sparse [12, 13]. Our rhinovirus findings are in agreement with a prior U.S. study on asymptomatic infants (0–5 months), which found overall rhinovirus prevalence of 11.4 % with a rhinovirus-A and -C predominance (rhinovirus-A 7.0 %, rhinovirus-B 0 %, and rhinovirus-C 4.4 %) [12]. However, the earlier study differed by including children with comorbidities such as wheezing, chronic heart–lung disease, and immunodeficiency. In addition, other community-based studies were conducted among infants at high risk for atopy, including a Dutch study that used nasal brush specimens and found a rhinovirus prevalence of 17 % among 6 month old asymptomatic infants, the majority of whom had a parent with allergic disease [5]. Likewise, in the Childhood Origins of Asthma study, the rhinovirus prevalence was 32 % using nasal washes among infants <1 year, all of whom were at high risk for atopy [5]. Additional studies among healthy, community-based infants age <1 year using nasopharyngeal swabs or nasal washes reported rhinovirus prevalence of 12–24 %, but based on small samples (n < 35 subjects) [7, 8]. A major strength of our study, compared to previous research, is assessing viral prevalence and rhinovirus species in a young population (median age 3.8 months, comparable to the median age of bronchiolitis hospitalization [4]) without comorbidities or high-risk of atopy.

Interestingly, we found that the rhinovirus genomic load among infants with rhinovirus detection was intermediate [14]. The mechanisms of rhinovirus positivity are unclear and likely multifactorial—e.g., active replication without respiratory symptoms, prolonged viral shedding, delayed clearance, and combinations of these factors in the population. The observed difference in patient characteristics (e.g. a higher rate of day care attendance and exposure to passive smoke in home among infants with rhinovirus detection) may paly a role in these potential mechanisms. The significance and mechanisms of rhinovirus detection in asymptomatic children merit further investigation.

Our finding of low RSV prevalence in asymptomatic infants suggests that RSV is likely the causative agent when detected in the setting of clinical symptoms of bronchiolitis. By contrast, given the observed prevalence of rhinovirus in infants without ARI, and even in infants without any respiratory symptoms, it remains unclear if rhinovirus detection always reflects current illness. Rhinovirus is frequently detected in the setting of multiple coexisting viruses [4]. Although a recent study of healthy infants showed that persistence of rhinovirus genome beyond 30 days was uncommon, 8.9 % of distinct rhinovirus infections lasted longer than 14 days, and 4.5 % lasted longer than 30 days [15]. Thus, while rhinovirus detected during bronchiolitis hospitalization can have an etiologic role, an alternate scenario is that the detected rhinovirus is persistent from a previous infection, and perhaps a marker of subject susceptibility or impaired immune response to rhinovirus infection. The generally higher rhinovirus prevalence found in studies of asymptomatic infants at high risk for atopy [5] may support this theory.

Our study has several potential limitations. First, the sample size, although larger than several prior studies, remains relatively small and all infants were recruited from one group practice in Boston, which may limit our generalizability to other settings. Second, this study was a single-year study; therefore, it is possible that an outbreak of a less virulent strain of rhinovirus occurred during this study period. Additionally, specimens were collected from late fall to early spring, yet rhinovirus infections occur year-round. While the observed prevalence could be different in months we did not enroll subjects, this chosen study period is helpful for bronchiolitis researchers. Third, ours study did not test other respiratory viruses, such as adenovirus, coronavirus, and human metapneumovirus. However, RSV and rhinoviruses are the most frequently detected respiratory viruses in young children (e.g., 85 % in children hospitalized for bronchiolitis [4]). Lastly, this cross-sectional study did not follow up the infants after enrollment to determine if some went on to develop ARI in the days after sample collection. Nevertheless, our results add to a surprisingly sparse literature and will aid interpretation of studies on viral bronchiolitis.

## Conclusions

In summary, in this cross-sectional study of healthy, community-based infants age < 1 year without ARI or comorbidities, RSV was rare (<2 %) in nasal swabs, while rhinovirus was detected in 11–15 % with a predominance of rhinovirus-A and -C. These finding are important for understanding the clinical significance of rhinovirus detection among infants hospitalized for bronchiolitis. To help tease out the role of rhinovirus detection in severe bronchiolitis and asthma pathogenesis, we plan to pursue future studies comparing rhinovirus prevalence, including genotyping, between infants hospitalized for severe bronchiolitis and healthy, community-based controls.

## Abbreviations

ARI: acute respiratory infection
PCR: polymerase chain reaction
RSV: respiratory syncytial virus

## References

[1] Hasegawa K, Tsugawa Y, Cohen A, Camargo CA (2015). Infectious disease-related emergency department visits among children in the US. Pediatr Infect Dis J.

[2] Hasegawa K, Tsugawa Y, Brown DF, Mansbach JM, Camargo CA (2014). Temporal trends in emergency department visits for bronchiolitis in the United States, 2006–2010. Pediatr Infect Dis J.

[3] Hasegawa K, Tsugawa Y, Brown DF, Mansbach JM, Camargo CA (2013). Trends in bronchiolitis hospitalizations in the United States, 2000–2009. Pediatrics.

[4] Hasegawa K, Mansbach JM, Camargo CA (2014). Infectious pathogens and bronchiolitis outcomes. Exp Rev Anti Infect Ther.

[5] Jartti T, Jartti L, Peltola V, Waris M, Ruuskanen O (2008). Identification of respiratory viruses in asymptomatic subjects: asymptomatic respiratory viral infections. Pediatr Infect Dis J.

[6] Chonmaitree T, Alvarez-Fernandez P, Jennings K (2015). Symptomatic and asymptomatic respiratory viral infections in the first year of life: association with acute otitis media development. Clin Infect Dis.

[7] Fry AM, Lu X, Olsen SJ (2011). Human rhinovirus infections in rural Thailand: epidemiological evidence for rhinovirus as both pathogen and bystander. PLoS One.

[8] Jansen RR, Wieringa J, Koekkoek SM (2011). Frequent detection of respiratory viruses without symptoms: toward defining clinically relevant cutoff values. J Clin Microbiol.

[9] Lee GM, Salomon JA, Friedman JF (2005). Illness transmission in the home: a possible role for alcohol-based hand gels. Pediatrics.

[10] Lu X, Holloway B, Dare RK (2008). Real-time reverse transcription-PCR assay for comprehensive detection of human rhinoviruses. J Clin Microbiol.

[11] Bochkov YA, Grindle K, Vang F, Evans MD, Gern JE (2014). Improved molecular typing assay for rhinovirus species A, B, and C. J Clin Microbiol.

[12] Iwane MK, Prill MM, Lu X (2011). Human rhinovirus species associated with hospitalizations for acute respiratory illness in young US children. Infect Dis J.

[13] Muller L, Mack I, Tapparel C (2015). Human rhinovirus types and association with respiratory symptoms during the first year of life. Pediatr Infect Dis J.

[14] Jartti T, Hasegawa K, Mansbach JM, Piedra PA, Camargo CA (2015). Rhinovirus-induced bronchiolitis: lack of association between virus genomic load and short-term outcomes. J Allergy Clin Immunol.

[15] Loeffelholz MJ, Trujillo R, Pyles RB (2014). Duration of rhinovirus shedding in the upper respiratory tract in the first year of life. Pediatrics.

